# An Efficient Image Compressor for Charge Coupled Devices Camera

**DOI:** 10.1155/2014/840762

**Published:** 2014-07-07

**Authors:** Jin Li, Fei Xing, Zheng You

**Affiliations:** Collaborative Innovation Center for Micro/Nano Fabrication, State Key Laboratory of Precision Measurement Technology and Instruments, Department of Precision Instruments, Tsinghua University, Beijing 100084, China

## Abstract

Recently, the discrete wavelet transforms- (DWT-) based compressor, such as JPEG2000 and CCSDS-IDC, is widely seen as the state of the art compression scheme for charge coupled devices (CCD) camera. However, CCD images project on the DWT basis to produce a large number of large amplitude high-frequency coefficients because these images have a large number of complex texture and contour information, which are disadvantage for the later coding. In this paper, we proposed a low-complexity posttransform coupled with compressing sensing (PT-CS) compression approach for remote sensing image. First, the DWT is applied to the remote sensing image. Then, a pair base posttransform is applied to the DWT coefficients. The pair base are DCT base and Hadamard base, which can be used on the high and low bit-rate, respectively. The best posttransform is selected by the *l*_*p*_-norm-based approach. The posttransform is considered as the sparse representation stage of CS. The posttransform coefficients are resampled by sensing measurement matrix. Experimental results on on-board CCD camera images show that the proposed approach significantly outperforms the CCSDS-IDC-based coder, and its performance is comparable to that of the JPEG2000 at low bit rate and it does not have the high excessive implementation complexity of JPEG2000.

## 1. Introduction

Charge coupled devices (CCD) camera is now heading for a high spatial resolution, high radiation resolution, large field of view, and wide coverage development [1–3]. In order to meet the performance of CCD camera, the CCD pixels number is growing, read-out rate is increasing, quantization bits of an analog-digital (AD) converter are increasing, and average shooting time is increasing. Therefore, the amounts of digitization image data in CCD camera are increasing sharply.  Table 1 shows input date rates of compression system for France earth observation satellites in recent years.

From Table 1, on-board image data rates are continuously increasing. However, the highest data transmission rates of on-board downlink channel are limited. In addition, the amount of flash-based nonvolatile solid state memory on the satellite is also limited. So, it is necessary to compress the on-board CCD images as well.

**Figure 1:**
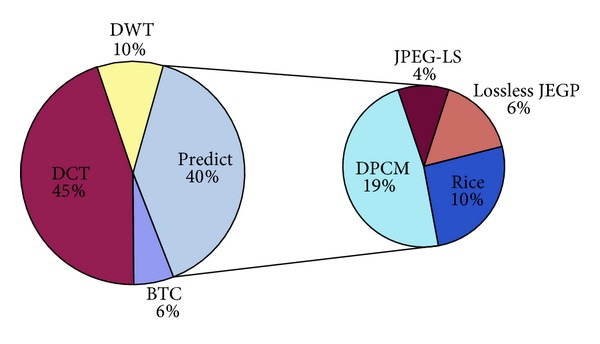
The statistics result of on-board image compression.

Space CCD camera compressor requires low complexity, high robustness, and high performance because of its captured images information being very precious, and also because it usually works on the satellite where the resources, such as power, memory, and processing capacity, are limited [4, 5]. Yu et al. [6] do statistics about the on-board image compression algorithm based on the basis compression theory used in compression system for more than 40 space missions. The statistics result is shown as in Figure 1. As is described in the picture, more than half of on-board image compression algorithms are based on a transform approach. For now, the most advanced on-board compression is based on the wavelet transform, which will also be the key technique in space camera compression application.

In recent years, many of discrete wavelet transforms- (DWT-) based compression approaches have been proposed, such as EZW [7], SPIHT [8], and SPECK [9]. The typical DWT-based algorithms are JPEG2000 [10] and the Consultative Committee for Space Data Systems-Image Data Compression (CCSDS-IDC) [11]. JPEG2000 algorithm is composed primarily of DWT and embedded block coding with optimal truncation points (EBCOT) [12]. JPEG2000 has the good compression results. However, JPEG2000 algorithm is too complex because three coding passes are required for each bit plane. In addition, the optimal rate control in JPEG2000 algorithm has high implementation complexity whereas the suboptimal rate control is inaccurate. This makes the implementation of JPEG2000 on space limited hardware particularly challenging. Therefore, The Consultative Committee for Space Data Systems (CCSDS) thinks JPEG2000 is not adapted to on-board compression. The CCSDS-IDC algorithm is composed of DWT and BPE. The BPE, which a zero-tree encoder, makes the most of the structures of spatiotemporal orientation trees in bit plane. That is, grandchildren coefficients also become not important when children coefficients are important. This zero-tree characteristic makes the bit plane exit a large amount of zero area. To take full advantage of these zero areas can improve coding efficiency. CCDS-IDC has progressive coding and fault-tolerant capability characteristic. But also, BPE has low complexity and occupies less storage capacity, which is very suitable for the application of on-board camera. However, it decreases the average PSNR by 2 dB compared with JPEG2000.

For remote sensing images having the abundant texture and edge features, DWT is not the optimal sparse representation [18–21], so that remote sensing images project on the DWT basis to produce a large number of large amplitude high-frequency coefficients, which are disadvantage for the later coding. In JPEG2000 algorithm, EBCOT [22] is very efficient in removing the redundancy between wavelet transforms coefficients, which makes JPEG2000 the best-performing compression encoder in the existing image compression algorithms. To overcome DWT issue, several promising transforms such as bandelets [23, 24], curvelets [25], contourlets [26], wedgelets [27], edgelets [28], and complex wavelet [29] have already been studied. However, these approaches usually require oversampling having higher complexity when compared to the wavelet and require nonseparable processing and nonseparable filter design.

Attempts on removing the redundancy between wavelets coefficients transform can be classified into two categories: one category transform in spatial domain and another category transform in transform domain. In [30], a two-dimensional (2D) edge adaptive lifting structure was presented. The 2D prediction filter predicts the value of the next poly phase component according to an edge orientation estimator of the image. In [31], Chang and Girod proposed an adaptive lifted discrete wavelet transform to locally adapt the filtering direction to the geometric flow in the image. In [32], a direction-adaptive DWT (DA-DWT) was proposed, which locally adapts the filtering directions to image content based on directional lifting. In [33, 34], oriented ID multiscale decomposition on a quincunx sampling grid was proposed, which obtains transform by adapting the lifting steps of an ID wavelet transform along local orientation. In [35, 36], adaptive directional lifting (ADL) was proposed, which performs lifting-based prediction in local windows in the direction of high pixel correlation. In [37, 38], weighted adaptive lifting- (WAL-) based wavelet transform was proposed, which uses the weighted function to make sure that the prediction and update stages are consistent. In [39], a 2D oriented wavelet transform (OWT) was introduced, which can perform integrative oriented transform in arbitrary direction and achieve a significant transform coding gain. However, these approaches usually produce blocking artifacts because the transform is performed in spatial domain.

To overcome this issue, Peyré et al. [40, 41] propose a new low-complexity compression approach based on posttransform (PT). PT is a transform to wavelet coefficients block. This approach can remove the redundancy between wavelets coefficients, which can improve compression performance. In addition, because it processes a 16-coefficient block and only carries out dot product operation, it does not require the large amount memories and could simply implement on hardware. The posttransforms destroy the zero-tree structure, so that the posttransformed coefficients are only encoded by entropy coding approaches, such as arithmetic coding, Huffman coding, not zero-tree coding approaches, such as BPE and SPIHT. However, on-board compression approaches require the embedded and progressive coding characteristics. To adapt on-board application, Delaunay et al. in [42–44] proposed a compression scheme using BPE from the CCSDS recommendation to code posttransform coefficients. However, they only apply the posttransform to the grandchildren coefficients, so that the compression performance is not that much better.

In this paper, we proposed a low-complexity posttransform coupled with compressing sensing (PT-CS) compression approach for remote sensing image. In the DWT domain, a pair base, DCT and Hadamard base being able to use, respectively, on the high and low bit-rate, the best posttransform is selected by the *l*_*p*_ *-*norm-based approach. The posttransform is considered as the sparse represent stage of CS. The posttransform coefficients are resampled by sensing measurement matrix.

The rest of this paper is organized as follows.  Section 2 introduces the proposed algorithm. In Section 3, the experimental results are demonstrated.  Section 4 concludes the proposed method.

## 2. Proposed Algorithm

### 2.1. The Imaging Principle of CCD

**Figure 2:**
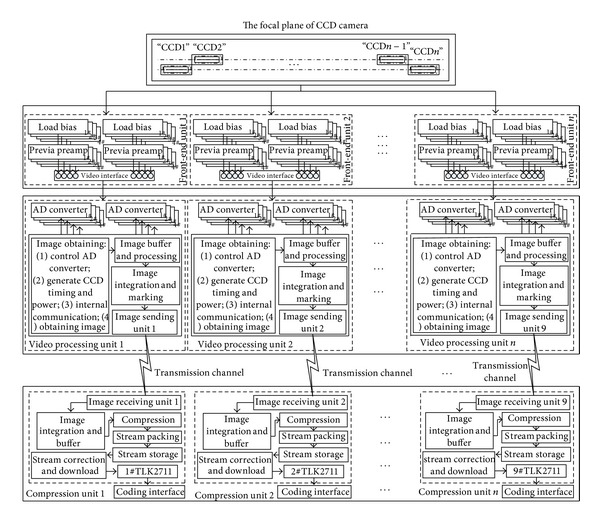
CCD image compression system.

In order to explain the background of efficient image compression system for CCD image data, this paper first briefly introduces the imaging principle of CCD camera. The general structure of the system is plotted in Figure 2. The system contains *n* CCDs. The pixel number of the panchromatic CCD is *N*, such as 12000. Moreover, The CCD has four channels of analog signal parallel outputs and 96 integral numbers. To avoid failure of function of the whole system due to single point, the analog video signal of each CCD is processed independently. So, the system needs mutually independent *n* image compression systems which compress image data of each CCD output. A 12-bit special video processor is used for each channel of each CCD. The calculation formula of the data rate of each CCD output image is

(1) $$\begin{array}{c} K_{\text{CCD}}=m\times n\times f_{L},\\ f_{L}=\frac{f}{H}\times \frac{V}{a}, \end{array}$$

where *m* is the number of CCD valid elements, *n* is the number of quantization bits, *f*_*L*_ is the push-broom line frequency, *f* is the focus of space cameras, *H* is the average height of satellite orbit, *V* is the subsatellite point velocity, and *a* is the CCD pixel size.

When *H* is 500 km, *f* is 3.5 m, and *V* is 7063 m/s, the line frequency of the CCD is 7.06 KHz. The total image data rate of four channel of CCD is 1.01664 Gbps. However, the data-transfer rate of on-board downlink channel is 300~600 Mbps. Let the CCD number *n* be equal to 4. In order to meet the task, our compressor requires the compression ratio at 4 : 1~32 : 1.

**Figure 3:**
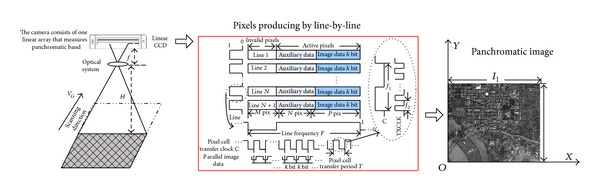
Imaging principle of CCD.

The general structure of the imaging principle of CCD is plotted in Figure 3. The long linear CCD consists of one linear array that measures the panchromatic spectra in the 0.4–0.9 region. The light radiated or reflected by the hundreds of kilometers of linear arrays of ground pixels is concentrated on optical thin film of CCD detector through an optical system. The space and spectrum distributing of ground targets radiation, acquired by CCD detector, can be expressed as

(2) B(x,y,θ,λ)=1π[E0(θ,λ)ρ(x,y,λ)τα(λ)∗hα(x,y,λ)]+N(θ,λ),

where *θ* is the solar height angle, *λ* is wavelength, *E*_0_ is ground luminometer, *ρ* is spectral reflectance, *τ*_*α*_ is atmospheric transmittances, *h*_*α*_ is the point spread function, and *N* is radiant flux. The optical thin film of linear array CCD allows the light of corresponding wavelength through. *B*(*x*, *y*, *θ*, *λ*) is captured by the linear CCD array to produce analog signals. Then, the analog signals are processed to produce one-line-image data. One-dimensional spatial information is gained. When the CCD camera scans the ground target, other spatial dimension information is gained. Therefore, CCD image is considered as a 2D image. Based on the imaging principle of CCD, CCD images have the spatial redundancies between the adjacent pixels. According to compression sensing (CS) sampling theory [45–47], sampling redundancy widely exists in images. In addition, a visual redundancy also exists in images. Therefore, the compression algorithm must remove spatial, sampling redundancy and visual redundancy efficiently. In order to meet the task, our compressor requires the PSNR greater than or equal to 35 dB at 4 : 1~32 : 1.

### 2.2. Spatial Decorrelation

The 2D DWT can decompose the image into lower resolution and detailed subband, which is viewed as successive low-pass and high-pass filtering. At each level, the high-pass filter produces detailed information called wavelet coefficients, while the low-pass filter associated with scaling function produces the approximate information called scaling coefficients. The DWT has been widely employed exploiting the spatial correlations for remote sensing image, such as JPEG2000 and CCSDS-IDC. In this paper, we apply a 2D DWT coupled with a posttransform to CCD image. In our approach, the 2D DWT is performed on CCD image, to reduce spatial correlations and then reduce remaining intraband correlations via a posttransform of the wavelet coefficients.

**Figure 4:**
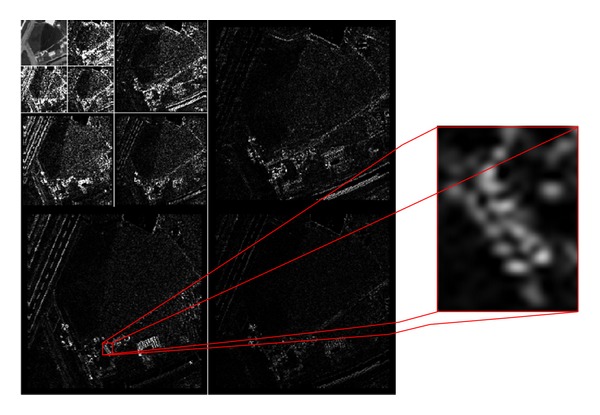
The wavelet transform of high resolution image with a zoom on some coefficients.

The 2D DWT has residual directional correlation between wavelet coefficients in a small neighborhood (see Figure 4). Statistical dependence between DWT coefficients has been studied for many years. In [48], correlations between nearby wavelet coefficients are reported in the range [0.01–0.54] at a distance less than or equal to 3 pixels. We found even wider range, and here, we provide a more detailed discussion regarding this topic. We use a Pearson correlation coefficient *ρ* [49] to analysis statistical dependency of DWT coefficients, which can be expressed as

(3) $$\begin{array}{c} \rho_{x,y}=\frac{\delta_{xy}}{\left(\delta_{x}\delta_{y}\right)}, \end{array}$$

where *δ*_*x*,*y*_ denotes the covariance between the variables *X* and *Y*; *δ*_*x*_ and *δ*_*y*_ denote the standard deviation of *X* and *Y*, respectively. According to project experience, three-level 2D DWT is appropriate for on-board compressor and we used three-level 2D DWT in this paper.

**Figure 5:**
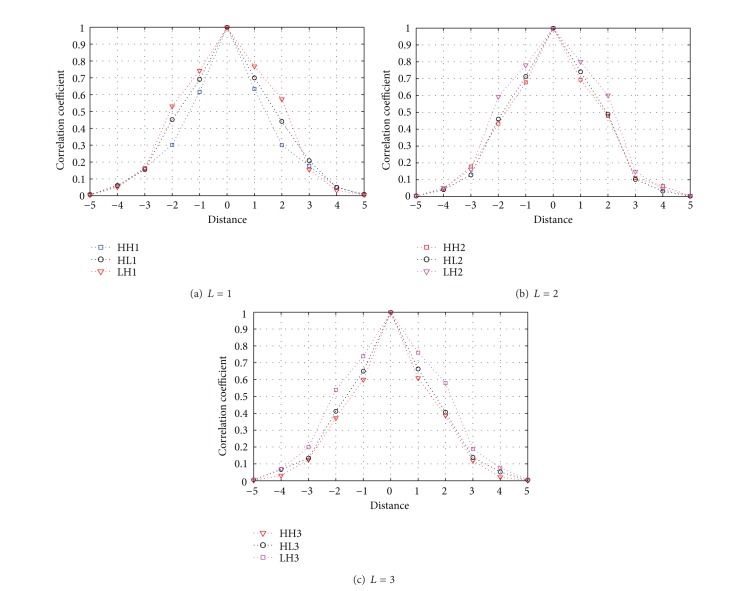
The correlation between wavelet coefficients in a small neighborhood.

Three-level 2D DWT is performed on each image band to produce one low-frequency subband (denoted by LL) and nine high-frequency subbands (denoted by *LH*⁡_*L*_, HL_*L*_, and HH_*L*_, *L* = 1, 2, 3). The test is performed from ten CCD images. The residual directional correlation between wavelet coefficients in a small neighborhood is shown as in Figure 5. In Level *L* (*L* = 1), the residual directional correlation within 16-connected region is *ρ*(HL1, HH1, *LH*⁡1, |Δ*x*| ≤ 2, |Δ*y*| ≤ 2) > 0.3. In Level *L* (*L* = 2), the residual directional correlation within 16-connected region is *ρ*  (HL2, HH2, *LH*⁡2, | Δ*x* | ≤2, | Δ*y* | ≤2) > 0.4. In Level *L* (*L* = 3), the residual directional correlation within 16-connected region is *ρ*  (HL3, HH3, *LH*⁡3, | Δ*x* | ≤2, | Δ*y* | ≤2) > 0.3. These imply a strong redundancy among neighboring 4 coefficients. The results in Figure 3 also indicate that the residual correlations between nearby wavelet coefficients are much weaker at a distance equal to 5 pixels.

For optimum compression performance of TDICCD image, algorithms must fully exploit the above-mentioned statistical properties. Several promising transforms such as contourlets, curvelets, ridgelets, and bandelets have been studied. However, their implementation complexity is too high. In [50], EBCOT has been reported to be very efficient in capturing these residual values. However, its implementation complexity is also too high. In [51], Delaunay has proposed a posttransform to exploit remaining redundancies between wavelet coefficients. After the wavelet transform of the image, posttransforms are applied on each block of 4 × 4 wavelet coefficients. This block size of posttransform is the best for simple and effective compression: the residual correlations between nearby wavelet coefficients are very low at a distance greater than and equal to 5 pixels, and the bigger the blocks, the more complex the computation; however when the block size decreases, the number of blocks and thus the side information increases. Note that no blocking artifacts are visible on the reconstructed image because the blocks are processed in the wavelet domain.

### 2.3. Posttransform Theory

This section gives a short review of posttransform, as introduced in [42, 44, 48]. The core idea behind posttransform compression is that wavelet coefficients blocks are further transformed using one group particular direction basis (such as bandelet, DWT, DCT, and PCA) in a dictionary. First, a 2D DWT is applied to an image. Next, blocks of 4 × 4 DWT coefficients are projected on *N*_*B*_ orthonormal bases *B*_*b*_ of the dictionary *D*. Then, a Lagrangian cost is computed and posttransformed coefficients are encoded. Each 4 × 4 DWT coefficients block *f* is considered as a vector of the space *R* ^*M*^ with *M* = 16. The *M* vectors of the basis *B*_*b*_ are noted *ϕ*_*m*_^*b*^ with *m* ∈ [1, *M* − 1]. The posttransformed block *f* ^*b*^ can be expressed as follows:

(4) $$\begin{array}{c} f^{b}=\overset{16}{\underset{m=1}{\sum }}\langle f,\phi_{m}^{b}\rangle \cdot \phi_{m}^{b}. \end{array}$$

Since a dictionary has *N*_*B*_ bases, *N*_*B*_ + 1 (including one original block) posttransformed blocks *f* ^*b*^ can be obtained. Among all the posttransformed blocks *f* ^*b*^, the best posttransformed block *f* ^b*^ is selected according to minimizing the Lagrangian rate-distortion cost. The minimizing of the Lagrangian rate-distortion cost can be expressed as

(5) $$\begin{array}{c} L\left(f_{q}^{b}\right)=D\left(f_{q}^{b}\right)+\lambda \cdot R\left(f_{q}^{b}\right), \end{array}$$

where *f*_*q*_^*b*^ denotes the quantized posttransformed coefficients, *q*is the quantization step, *D*(*f*_*q*_^*b*^) denotes the square error due to quantization of the posttransformed block *f* ^*b*^, *λ* is a Lagrangian multiplier, and *R*(*f*_*q*_^*b*^) denotes an estimate of the bit rate required for encoding *f*_*q*_^*b*^ and the associated side information b*.

### 2.4. Our Posttransform Method

In the posttransform, the dictionary has multiple bases. The better the compression performance is, the larger the number of bases is, and the higher the computational complexity is. However, on-board compression requires low computational complexity. The space CCD compressor allows only a one basis posttransform. In [52], a low-complexity compression scheme using the posttransform in only Hadamard basis has been proposed and the posttransform is applied only at the first level of the wavelet transform. The PSNR increase has been reported to be only between 0.4 dB and 0.6 dB compared to the DWT alone. In this paper, to obtain a low complexity yet efficient posttransform, we thus consider a very simple dictionary containing only one dynamic base, which is Hadamard basis at the low bit rates and DCT basis at the high bit rates.

In [53], Delaunay et al. have shown that the Lagrangian approach to select the best posttransformed block has two main drawbacks: (1) the bit rate estimation of encoding *R*(*f*_*q*_^*b*^) is computationally intensive and not always accurate; (2) the choice of the best posttransformed block depends on the quantization step q in the rate-distortion criterion. However, the coder does not define the quantization step when coding. Therefore, Delaunay et al. proposed an *l*_1_-norm minimization approach to select the best posttransformed block. In this paper, we propose a new method based *l*_*p*_-norm minimization approach, which replaces an *l*_1_-norm minimization method. It is adapted to the space TDICCD compressor constraints of low complexity.

At the low bit rate, the bit rate *R* can be expressed as

(6) $$\begin{array}{c} R\approx \gamma_{0}M\;\text{with}\;\;\gamma_{0}=7, \end{array}$$

where *M* is the number of nonzero posttransformed coefficients. We propose an *l*_0_-norm (*p* = 0) minimization approach to select the best posttransformed block. The selected best posttransformed block is the one with the fewest high magnitude coefficients:

(7) fb∗=arg min⁡fb,b∈[0,NB]||fb||0||fb||0=∑m=1MIm with  Im={1,⌊|ab[m]|⌋≠0,0,⌊|ab[m]|⌋=0,

where *a*_*b*_[*m*] is a posttransformed coefficient. At the high bit rate, the bit rate *R* can be expressed as

(8) $$\begin{array}{c} R\approx -\overset{N}{\underset{i=1}{\sum }}p_{i}\;\text{lo}{\text{g}}_{2}p_{i}-\text{lo}{\text{g}}_{2}q. \end{array}$$

We use the *l*_1_-norm (*p* = 1) minimization approach to select the best posttransformed block. The selected best posttransformed block is the one with the fewest high magnitude coefficients:

(9) $$\begin{array}{c} f^{b^{*}}=\underset{f^{b},b\in [0,N_{b}]}{\arg\;\min}{||f^{b}||}_{1}\;\text{s}.\text{t}.\;\;{||f^{b}||}_{1}=\left(\overset{15}{\underset{m=0}{\sum }}\left|a_{b}\left[m\right]\right|\right). \end{array}$$

**Figure 6:**
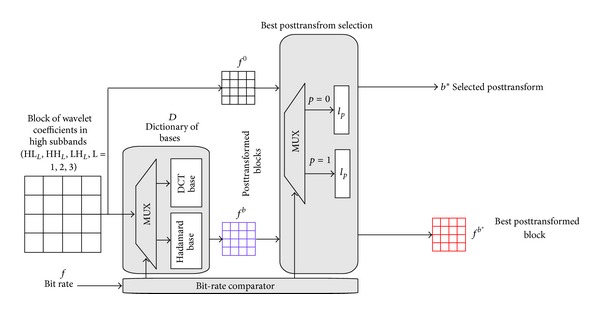
The proposed posttransform architecture.

Figure 6 shows the proposed posttransform architecture. Each high-frequency subband is performed by posttransform. The bit-rate comparator decides the type of coding bit rate. We define the coding bit rate to be greater than and equal to 0.5 bpp as the type of high bit rate (type 1) and less than 0.5 bpp as the type of low bit rate (type 0). The posttransform is performed using DCT basis and *l*_1_-norm minimization when coding type 1, Hadamard basis and *l*_0_-norm minimization when coding type 0.

### 2.5. Proposed Posttransform Compressing Sensing (PT-CS)

In [54], an adaptive arithmetic coder is used to encode both the posttransformed blocked coefficients and the side information of the chosen posttransform bases on each block. In [53], the bit-plane encoder (BPE) is used to encode both the posttransformed blocked coefficients and the side information. However, the posttransforms destroy the zero-tree structure; the PSNR using BPE is 0.2 dB less than using DWT and even worse at high bit rates. A basis vector ordering approach has been used. This ordering is defined by processing several thousand blocks of wavelet coefficients from a learning set of images. The ordering approach has two drawbacks for space CCD compression. First, the ordering for the basis vector is computationally extensive and not always accurate. Second, the ordering highly depends on a learning set of images learning. However, the learning set of images is not captured when coding.

Indeed, after 2D DWT and posttransform, the TDICCD image has been sparse in posttransform domain. In order to achieve a higher compression ratio and the low complexity yet, we consider the 2D DWT and posttransform as the sparse representation stage for the TDICCD image, and thus the posttransform coefficients can be resampled using sensing matrices to achieve compression. According to compressed sensing (CS), the sparse signal with a few significant samples in one basis can be reconstructed almost perfectly from a small number of random projections onto a second basis that is incoherent with the first.

First, the following gives a short review of CS, as introduced in [55]. A good overview can be found in [56]. A complete CS involves three main stages: sparse representation, sensing measurements, and signal reconstruction. The sparse representation stage is to represent the original signal *f* ∈ *R* ^*N*^ with a number of coefficients *x* = [*x*_1_,…, *x*_*N*_] in an *N* × *N* orthonormal transform basis matrix Ψ:

(10) $$\begin{array}{c} f=\Psi x=\overset{N}{\underset{i=1}{\sum }}x_{i}\varphi_{i}\;\text{with}\;\;x_{i}=\langle f,\varphi_{i}\rangle . \end{array}$$

If the number of nonzero or significant coefficients in the vector *x* is *K*, the original signal *f* is defined as *K*-sparsity in the Ψ basis. The sensing measurements stage is to project an original signal *f* into a cluster of measurements *y* with significantly less elements than *f*:

(11) $$\begin{array}{c} y=\Phi f\;\text{with}\;\;y_{i}=\langle f,\phi_{i}\rangle ,\;\;i\in \left[1,M\right], \end{array}$$

where Φ is an *M* × *N* measurement matrix. Then, the above expression can be written as

(12) $$\begin{array}{c} y=\Phi \Psi x=\Phi^{\prime }x. \end{array}$$

Since *M* ≤ *N*, compression can be achieved. Indeed, the core idea of CS is to remove sampling redundancy by requiring only *M* samples of the signal. The resultant measurements *y* are used for the recovery of original signal *f*. If the measurement matrix Φ is properly designed, ΦΨ can satisfy the so-called restricted isometry property (RIP). And the original signal *f*could be exactly or approximately recovered by solving the following standard convex optimization problem:

(13) $$\begin{array}{c} \tilde{x}=\arg\;\min{||x^{\prime }||}_{1}\;\text{s}.\text{t}.\;\;\Phi \Psi x^{\prime }=y. \end{array}$$

**Figure 7:**
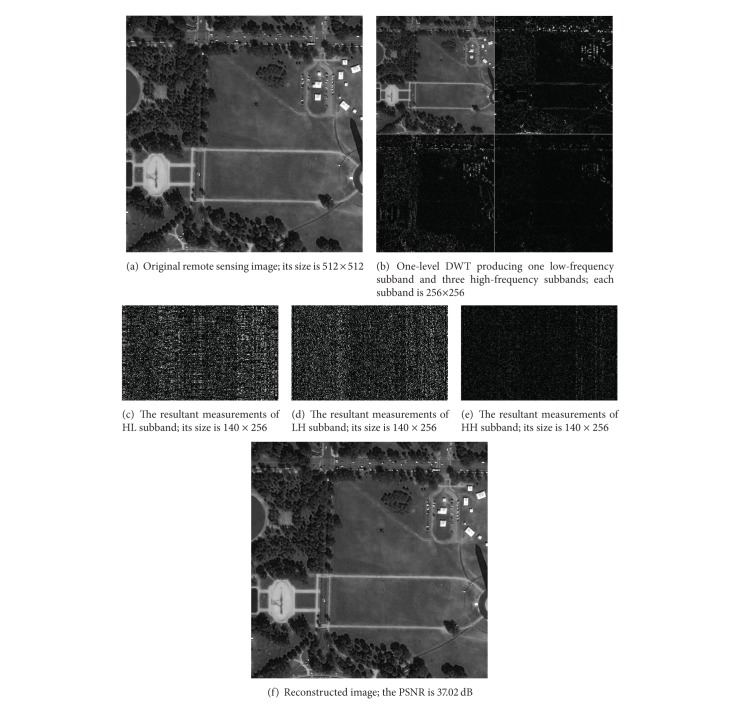
The compressive sensing process using DWT sparse representation for remote sensing.

In this paper, we apply CS to compress remote sensing image.  Figure 7 shows the compressive sensing process using DWT sparse representation for remote sensing. The remote image is 512 × 512. The DWT can produce one low-frequency subband, LL, and three high-frequency subbands, HL, LH, and HH. Each high-frequency subband performs sensing measurements using 140 × 256 Gaussian random measurement matrix. Since *M* ≤ *N*  (*M* = 140  and  *N* = 256), compression can be achieved. For three high-frequency subbands, the sparsity is 113, 71, and 7, respectively. The measurement numbers *M* of all high-frequency subbands satisfy *M* ≥ *K* × log⁡(*N*/*K*). The high-frequency subband coefficients can be reconstructed. The inverse DWT is performed to obtain reconstructed image. The PSNR of reconstructed image can reach 37.02 dB. Therefore, the CS offers better compression performance for remote sensing image.

In this paper, we consider the posttransformed coefficients as the *x*. Let Ψ_*W*_ be a DWT orthonormal basis matrix and let Ψ_*P*_ be a posttransform orthonormal basis matrix. Ψ_*W*_Ψ_*P*_ is considered as Ψ. So, the posttransformed coefficients perform the sensing measurements using measurement matrix to achieve compression. In CS sense, the posttransformed coefficients achieve compression by removing sampling redundancy in place of the BPE coder.

**Figure 8:**
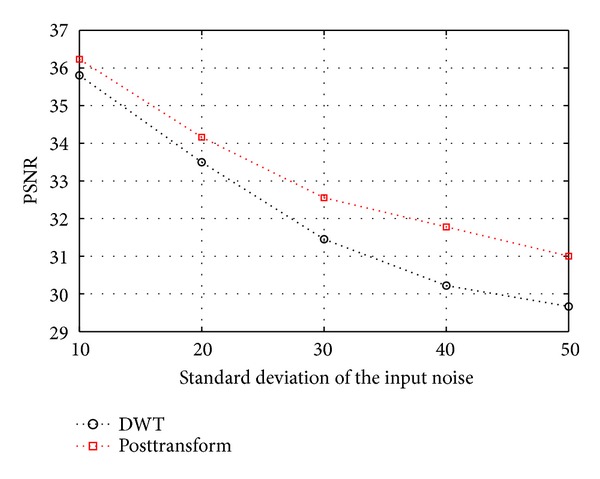
Denoising performance with both DWT and posttransform method.

In [57], wavelet-based CS has been proposed. The wavelet-based CS is considered for images are sparse in a wavelet basis. In our approach, we use DWT and posttransform as the transform basis at sparse representation stage. After image sparse representation, most of the image information is concentrated on small transform coefficients in *x*, and most of the transform coefficients in *x* are not zero but very small. We use the hard threshold- (HT-) based image denoising to indirectly measure the sparsity of transformed image since the better the sparsity is, the more the significant coefficients are after HT and the higher the peak signal-to-noise ratio (PSNR) of denoised image is. We use AVIRIS images in our test and choose the same threshold *T* as [58].  Figure 8 shows the PSNR results for the various transform approaches used in our method. As the picture shows, the posttransform offers better sparsity. This is because the posttransform can exploit remaining redundancies between wavelet coefficients.

**Figure 9:**
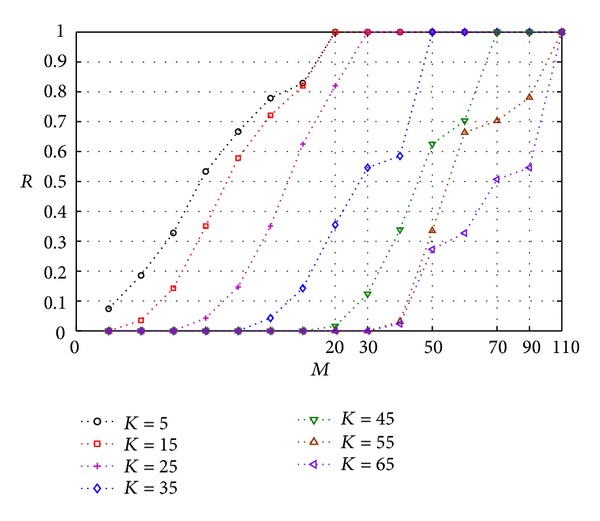
Variation trend between the sparsity *K* and measurement number *M*.

In CS system, the sparsity of transformed image is one of the key factors affecting reconstructed image quality. Below, we analyze the relationship between the number of measurement matrix *M* and the sparsity of transformed image *K*. We use Gaussian random matrix as sensing matrix. First, we study the relationship between *M* and *K* for one-dimension (1D) signal. We use a 1D signal with 256 samples and orthogonal matching pursuit (OPM) method to recover original signal. Assume *R* denotes the ratio of correct data numbers of recovery signal to all data numbers of original signal.  Figure 9 shows the variation trend between the sparsity *K* and measurement number *M*. To accurately recover original signal, the more *K* is, the more *M* is. And when *M* is higher than some threshold, the signal can be accurately recovered. That is, the better the sparsity of signal is, the less the measurement number *M* is, and the better the performance of compression and reconstructed signal quality are.

**Figure 10:**
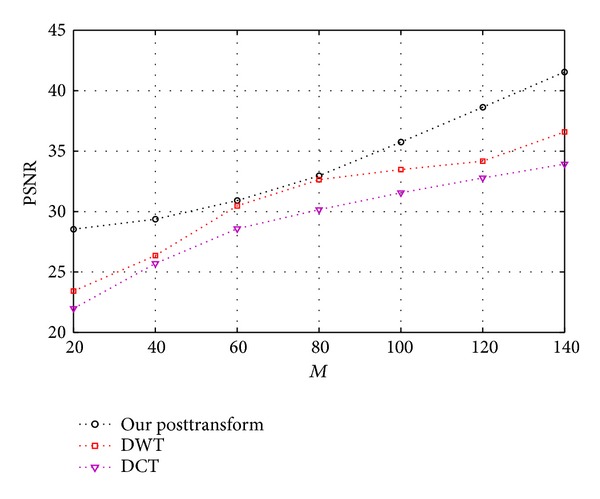
PSNR variation trend with measurement number *M* when using three bases.

**Figure 11:**
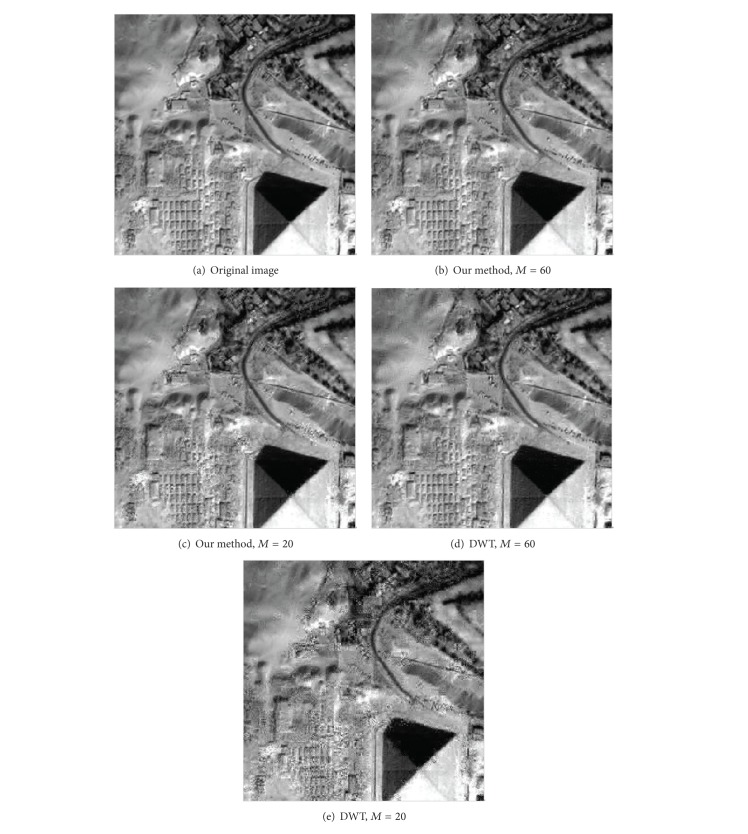
Reconstructed image using our method and DWT as the sparse basis when *M* = 20 and *M* = 40; the size of each image is 256 × 256.

For 2D remote images, we consider the DWT, DCT, and our posttransform as the sparse basis. All sparse bases lead to the PSNRs for the same *M* value (see Figures 10 and 11). The better the sparsity of image is, the better the reconstructed image quality is. Since our posttransform offers a better sparsity than wavelet-based transform, our posttransform as CS sparse representation stage is very suitable.

### 2.6. Deep Coupling between CS and Posttransform

**Figure 12:**
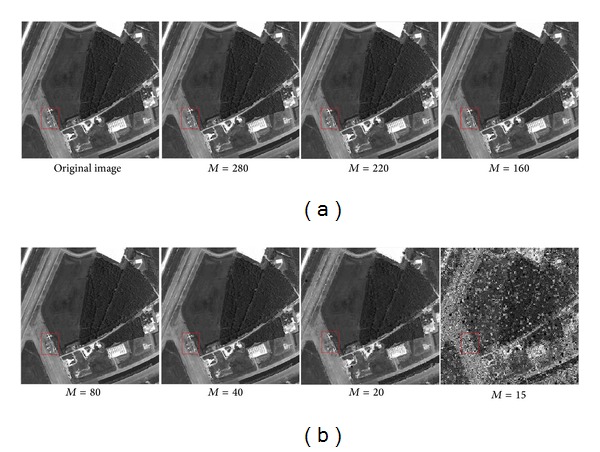
Reconstructed image using our method at different *M*; the size of each image is 512 × 512.

The sensing measurement number *M* exercises a great influence on reconstructed signal quality (see Figures 10 and 12) when using the posttransform basis. The more *M* is, the better reconstructed image quality is. The measurement number *M* of sensing matrix is determined by compression ratio (CR). The lower values of *M* give the higher compression ratio.

In our approach, each subband performs sensing measurement independently. The measurement numbers for subbands after posttransform, that is, LH_*L*_, HL_*L*_, and HH_*L*_  (*L* = 1, 2, 3), are denoted by *M*_LH_*L*__, *M*_HL_*L*__, and *M*_HL_*L*__  (*L* = 1, 2, 3). The result measurement can be expressed as

(14) $$\begin{array}{c} Y_{\text{L}{\text{H}}_{L}}^{\left(I_{1}/2^{L}\times M_{\text{L}{\text{H}}_{L}}\right)}=\Phi_{\text{L}{\text{H}}_{L}}^{\left(M_{\text{L}{\text{H}}_{L}}\times I_{2}/2^{L}\right)}\times X_{\text{L}{\text{H}}_{L}}^{\left(I_{2}/2^{L}\times I_{1}/2^{L}\right)},\\ Y_{\text{H}{\text{L}}_{L}}^{\left(I_{1}/2^{L}\times M_{\text{H}{\text{L}}_{L}}^{}\right)}=\Phi_{\text{H}{\text{L}}_{L}}^{\left(M_{\text{H}{\text{L}}_{L}}^{}\times I_{2}/2^{L}\right)}\times X_{\text{H}{\text{L}}_{L}}^{\left(I_{2}/2^{L}\times I_{1}/2^{L}\right)},\\ Y_{\text{L}{\text{H}}_{L}}^{\left(I_{1}/2^{L}\times M_{\text{H}{\text{H}}_{L}}^{}\right)}=\Phi_{\text{H}{\text{H}}_{L}}^{\left(M_{\text{H}{\text{H}}_{L}}^{}\times I_{2}/2^{L}\right)}\times X_{\text{H}{\text{H}}_{L}}^{\left(I_{2}/2^{L}\times I_{1}/2^{L}\right)}. \end{array}$$

The CR can be considered as the ratio between the total number of bits in the original transformed coefficients and the number of bits that must be transmitted, which can be expressed as

(15) CR=I1×I2×nB,B=∑L=13(∑i=1I1/2L ∑j=1MLHLnyLHL(i,j)+∑i=1I1/2L ∑j=1MLHLnyLHL(i,j)+∑i=1I1/2L ∑j=1MLHLnyLHL(i,j)),

where *n* denotes the bit depth of each pixel.

**Figure 13:**
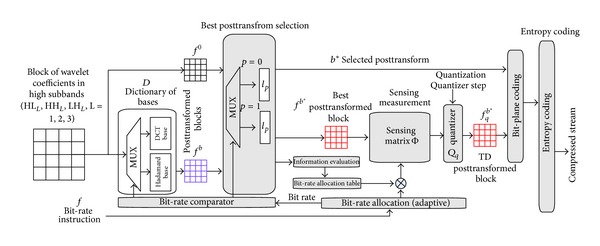
Proposed deep coupling algorithm.

In order to efficiently determine the measurement number *M* of all measurement matrices, we proposed a deep coupling between posttransform and CS to not only determine the measurement number *M* and reduce the side information of posttransform but also to code these measurement results and complete the bit-rate control.  Figure 13 shows the proposed deep coupling between the posttransform and CS.

The bit-rate allocation module allocates the bit rates for each sensing matrix. The information evaluation module evaluates the information of each tensor. Now the target bit rates for different sensing matrixes can be allocated based on their information contents. Then, the measurement number of each sensing matrix can be determined according its allocated bit rates.

In the first place, the information content in LL subband *I*_LL_ is calculated through an *l*_1_-norm approach. Let *f*(*x*, *y*) denote the coefficients in LL subband. The information content in LL subband can be calculated as

(16) $$\begin{array}{c} I_{\text{LL}}={||f_{\text{LL}}||}_{1}=\overset{I_{1}/8}{\underset{x=1}{\sum }}\;\;\overset{I_{2}/8}{\underset{y=1}{\sum }}\left|f\left(x,y\right)\right|. \end{array}$$

In the second place, the information content of 9 tensors (*LH*⁡_*L*_′, HL_*L*_′, HH_*L*_′, *L* = 1,2, 3) is calculated. Let *I*_*LH*⁡_*L*_′_, *I*_HL_*L*_′_, and *I*_HH_*L*_′_, *L* = 1,2, 3, denote the information content of 9 tensors, respectively. Since the selected representation *f* ^b*^ reflects the image information, the information content of 9 tensors can be evaluated through  *f* ^b*^. The dimensions of each tensor are *I*_1_ × 2^−*L*^ × *I*_2_ × 2^−*L*^ × *I*_3_. Each tensor contains *I*_1_ × 2^−*L*−2^ × *I*_2_ × 2^−*L*−2^ posttransformed blocks. Let *f*_*i*_^b*^ denote the selected representation of *i*th posttransform block. The information content of the tensor *LH*⁡_*L*_′ can be calculated as

(17) $$\begin{array}{c} I_{{\mathrm{LH}}_{L}^{\prime }}=\overset{I_{1}\times I_{2}\times 2^{-2(L-2)}}{\underset{i=1}{\sum }}f_{i}^{b^{*}}. \end{array}$$

Other tensors can be likewise calculated.

In the third place, the weight of bit-rate allocation for each tensor is acquired through

(18) $$\begin{array}{c} r_{{\mathrm{LH}}_{L}^{\prime }}=\frac{I_{{\mathrm{LH}}_{L}^{\prime }}}{I_{\text{LL}}+\sum_{L=1}^{3}{\mathrm{LH}}_{L}^{\prime }+{\text{HL}}_{L}^{\prime }+{\text{HH}}_{L}^{\prime }},\\ r_{{\text{HL}}_{L}^{\prime }}=\frac{I_{{\text{HL}}_{L}^{\prime }}}{I_{\text{LL}}+\sum_{L=1}^{3}{\mathrm{LH}}_{L}^{\prime }+{\text{HL}}_{L}^{\prime }+{\text{HH}}_{L}^{\prime }},\\ r_{{\text{HH}}_{L}^{\prime }}=\frac{I_{{\text{HH}}_{L}^{\prime }}}{I_{\text{LL}}+\sum_{L=1}^{3}{\mathrm{LH}}_{L}^{\prime }+{\text{HL}}_{L}^{\prime }+{\text{HH}}_{L}^{\prime }}. \end{array}$$

In this paper, the bit-plane coding procedure is used to code the quantized measurement results. We modify the SPITH algorithm [59] to complete the bit-plane coding. The bit-plane coding processes each quantized measurement result at a time. After one quantized measurement result is processed, the next quantized measurement result is processed. For each time, the bit-plane coding includes two passes: the significant pass (SP) and the refinement pass (RP). We define a significant map of a given threshold *T* and the quantized measurement result element *g*_*j*_1_*j*_2_*j*_3__ at the location (*j*_1_, *j*_2_, *j*_3_). Let *S*_*n*_(*T*) be the significant state for the threshold *T* (where *T* is an *n* power of 2) in the *n*th bit plane; for example,

(19) $$\begin{array}{c} S_{T}\left(n\right)=\{\begin{array}{cc} 1, & T\leq \left|g_{j_{1}j_{2}j_{3}}\right|\leq 2T,\\ 0, & \text{otherwise}. \end{array} \end{array}$$

For *S*_*T*_(*n*) = 1, the *g*_*j*_1_*j*_2_*j*_3__ is considered as the significant element. The significant element must be encoded and removed from the quantized measurement result. The insignificant elements are preserved for the next bit plane. After that, the significant threshold is divided in half, and the process is repeated for the next pass.

### 2.7. Our Proposed Codec Architecture

**Figure 14:**
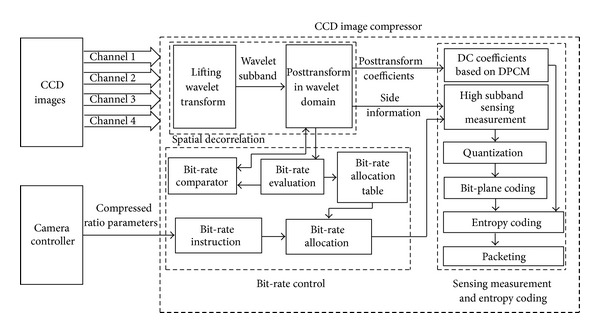
Compression algorithm architecture for CCD image using the proposed PT-CS method.

In order to remove spatial, sampling redundancy and visual redundancy efficiently, we proposed a low-complexity posttransform coupled with compressing sensing (PT-CS) compression approach for remote sensing image.  Figure 14 shows the proposed architecture. The compression is performed using four steps. In the first step, 3-level 2D DWT is applied to the TDICCD image to obtain one low-frequency subband and 9 high-frequency subbands. In the second step, blocks of 4 × 4 DWT coefficients of all the subbands except the LL subband are posttransformed. In the third step, the CS is applied to the subbands. In the fourth step, the posttransformed coefficients in low-frequency subband are encoded by DPCM. The measurement results perform quantization and the bit-plane coding and then entropy coding using adaptive arithmetic coding. In the fifth step, the bit streams are packed and transmitted to the decoder via signal channels.

**Figure 15:**
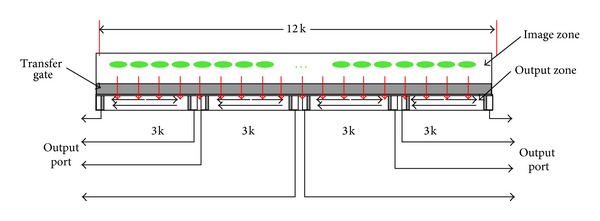
The outline structure of the linear CCD.

Note that CCD outputs four-channel images. The four-channel images is processed simultaneously by the PT-CS compressor. Therefore, the synchronicity of the four-channel images is very important and will affect the compression performance of the proposed algorithm. In fact, the linear CCD produces 12 k pixels (see Figure 15), which is output by four channels or eight channels. In our system, we use the four-channel way. Image zone produces charges and the charges are moved to the output zone. In output zone, charges are read out by four channels. In order to avoid the pixel smear, charges in output zone must be read out by four channels and simultaneously by diver clocks before charges in image zone moving to output zone. Therefore, the four-channel image is always output simultaneously.

## 3. Experimental Results

### 3.1. Experimental Scheme

**Figure 16:**
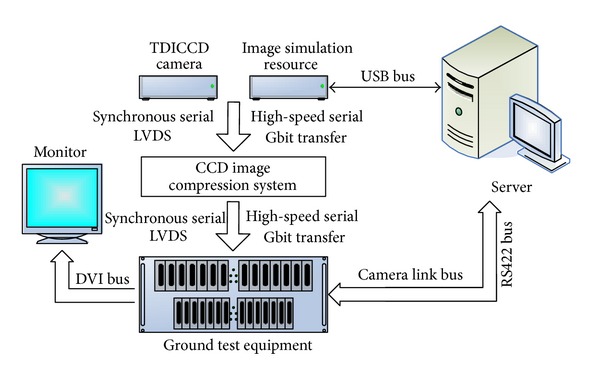
The structure of experiment system.

**Figure 17:**
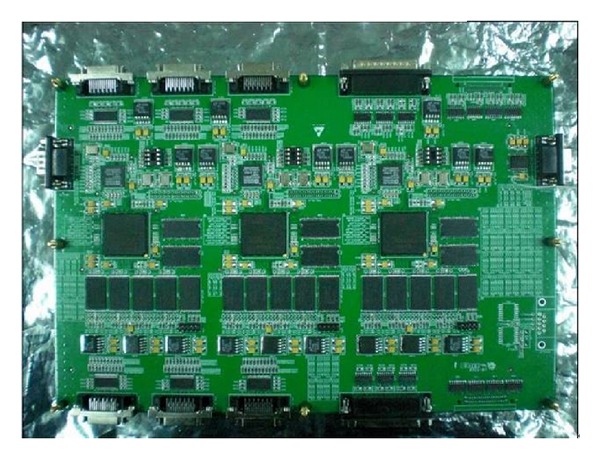
The designed compression system for CCD camera using our PT-CS algorithm.

In order to test the performance of the proposed algorithm in this paper, we use the independent development ground test equipment. The experimental system used to test the performance of the proposed algorithm is shown in Figure 16. The experiment system is composed of TDICCD camera, image simulation resource, TDICCD image compression system, ground test equipment, DVI monitor, and server. The TDICCD image compression system is shown as in Figure 17. The server injects the remote sensing image into the image simulation resource, which adjusts the images to simulate the output of CCD and then transfers the images to TDICCD image compression system to verify the compression algorithm. The ground test equipment implements the image decompression to gain the reconstructed images and then transfers them to the server through camera link bus. Finally, the compression performance is analyzed in the server.

### 3.2. TDICCD Image Compression Validation and Analysis

**Figure 18:**
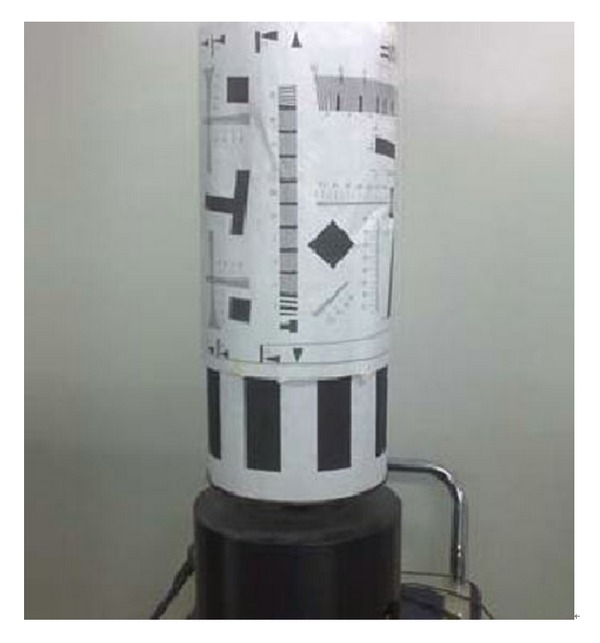
The rotating cylinder as a target.

**Figure 19:**
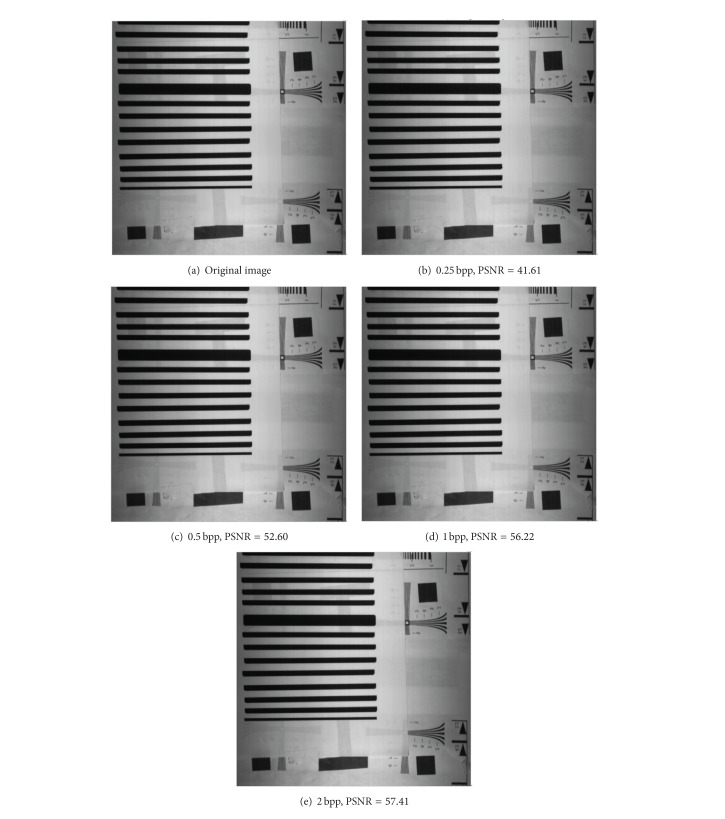
Reconstructed image at different bit rate.

In order to verify the validation of the proposed algorithm, we perform two step experiments. In the first place, CCD camera sends test image to compression system using the rotating cylinder as a target (see Figure 18). CCD camera captures rotating cylinder target to produce test image. CCD outputs image by line to line. The working line frequency is set to 7.06 KHz. The pixels number of each line image is 3072. Within the camera shooting 15 minutes, the captured images store the NAND flash array in the camera. 20-frame image from the NAND flash array is sent to compression system. Each frame image is 1000 × 1000, the bit depth per pixel is 10 bit. The test image is encoded, transferred by high-speed serial G-bit transfer system, reconstructed by ground test equipment, and then sent to PC computer through Camera Link.  Figure 19 demonstrates the reconstructed images at different bit rates.

**Figure 20:**
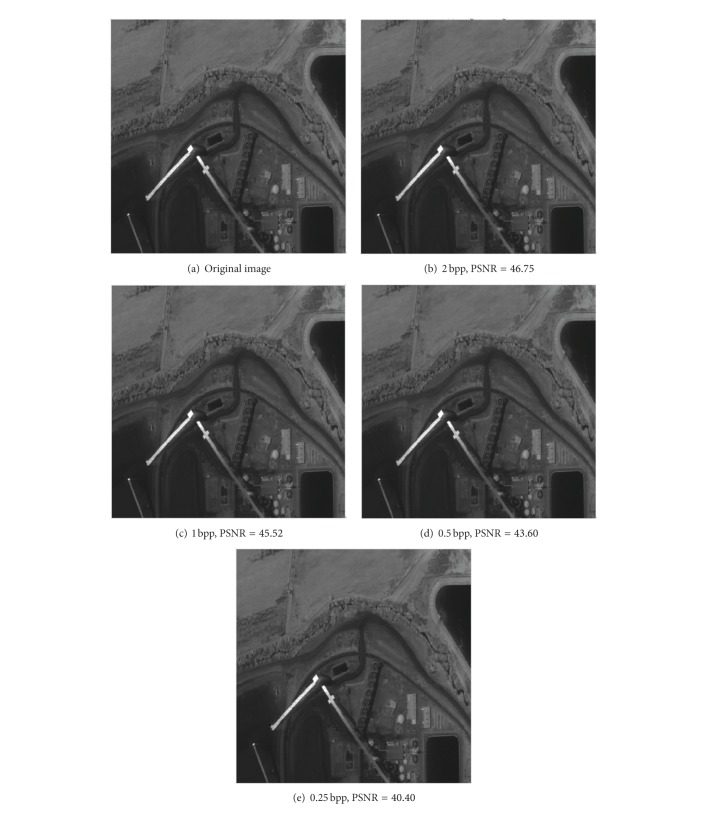
Reconstructed remote image at different bit rate.

In the second place, the server injects the AVIRIS multiband remote sensing images into the image simulation resource. The number of test remote sensing images is six. The size of images is 512 × 512, 8 bpp (bits per pixel). The compression rate CR is set to CR = 8/1 = 8.  Figure 20 demonstrates the reconstructed remote sensing images at different bit rates. From the displayed images, the original image and reconstructed image have almost no difference because the proposed compression algorithm has a high signal-to-noise ratio. The PSNR reaches 46.75 dB, 45.52 dB, 43.60 dB, and 40.40 dB, respectively, in Figures 20(a)~20(e). After each image was encoded by the proposed algorithm at 1 bpp, the dynamic range of pixels is 0~2 bit, and the value of most pixels are about 1 bit. So, the proposed algorithm is validation for space CCD image compression.

### 3.3. Remote Sensing Image Compression Algorithm Performance Analysis

To objectively evaluate the performance of proposed deep coupling-based compression scheme, extensive experiments were carried out on a number of multispectral data at various coding bit rates. In the first experimental part, in order to test the compressed performance of proposed approach, we use three groups of SPOT-1 remote images having different texture characteristics. The quality assessment of the decoded images is based on rate-distortion results measured by means of the overall SNR given by

(20) $$\begin{array}{c} \text{PSNR}=10\;\text{lo}{\text{g}}_{10}\left(\frac{P}{\text{MSE}}\right)\left(\text{dB}\right), \end{array}$$

where *P* and MSE denote the power of the original image and the mean squared error, respectively.  Table 2 demonstrates the tested PSNR results of our approach at different bit rates.

According to Table 2, the average PSNR reaches up to 40 dB, so the proposed algorithm has well compressibility and satisfies the requirements of design index.

**Figure 21:**
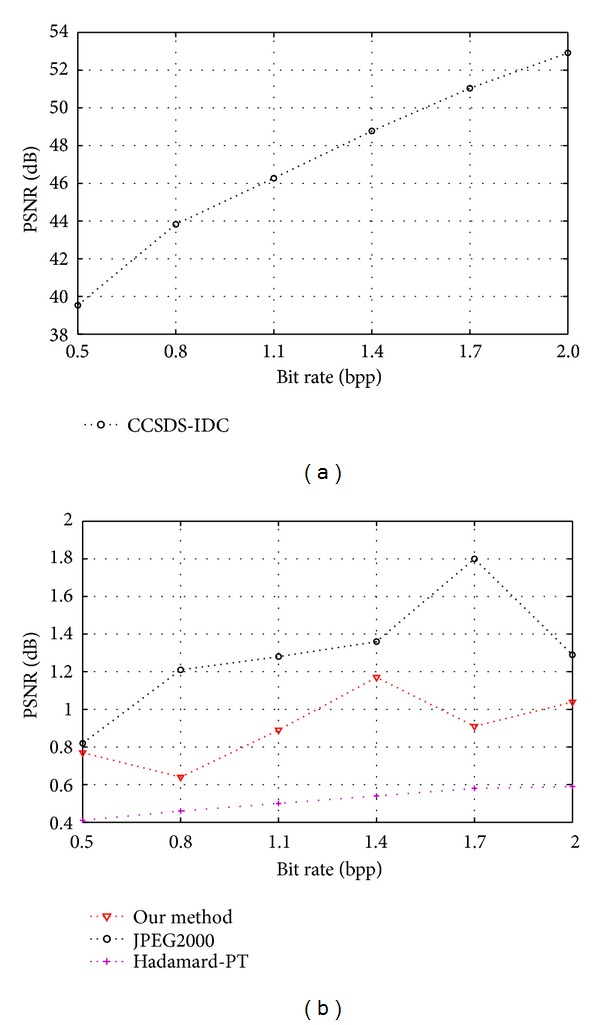
The test and comparison result of multiband images, the left is PSNR of 3 compression codec, and the right is PSNR difference between CCSDS with other 2 compression codecs.

In the second experiment, in order to compare compressed performance of our approach with other algorithms, we compare the compression results obtained with the proposed coder against those achieved with CCSDS-IDC, JPEG2000, and Hadamard posttransform implemented independently. We use the AVIRIS remote sensing images in JPL laboratory. Each group is 512  pixel × 512  pixel, the depth of every pixel is 8 bit. The images are Low Altitude, Lunar Lake, Jasper Ridge, and Cuprite. Each test remote sensing images number is three. The compression rate is set to 2.0~0.25 bpp. The average PSNR comparisons were demonstrated in Figure 21. From Figure 21, due to the full usage of posttransform and CS the proposed compression scheme achieves the higher compression performance and 0.3~1.3 dB PSNR gain on average against CCSDS-IDC and Hadamard posttransform compression codec in norm bit rate 2.0~0.25 bpp. And our approach is only lower (0.1~0.9 dB) than JPEG2000. Overall, the proposed scheme shows excellent lossy compression performance and delivers better compression results than that of commonly used coders.

### 3.4. Proposed Algorithm Complexity Analysis and Compression Time

In the following, we analyze the complexity of the algorithm. In our method, three-level 2D DWT is applied to the spatial bands. We considered an *N*-tap filter bank and denoted by *L* the number of wavelet decomposition levels in the spatial band. The complexity of applying 2DWT to multispectral images with size of *I*_1_ × *I*_2_ × *I*_3_ is *O*(8*NI*_1_ *I*_2_ *I*_3_(1 − 2^−2*L*^)/6). In our method we use 9/7 DWT and also three levels of decomposition, so the complexity of our algorithm is *O*(9*I*_1_ *I*_2_ *I*_3_/7). After applying 2D DWT, in low bit rate, we use the Hadamard posttransform. For each block of *M* coefficients, the Hadamard transform needs the *O*(*M*log⁡*M*) operations. In high bit rate, we use the DCT posttransform. It is pointed out that the calculation load of DCT is much less than that of DWT. One of the most efficient ways for 2D 8 × 8 DCT requires only 54 multiplications and 462 additions. It means that each point needs merely 0.84 multiplications and 7.22 additions. Therefore, 2D 4 × 4 DCT requires only 27 multiplications and 231 additions. Then, the best posttransform selection requires 2 × (*M* − 1) additions and one comparison. So, the complexity required is *M*(log⁡_2_ *M* + 3) + 2 operations per block of *M* coefficients.

After applying posttransform, the multiplication complexity of sensing measurement sensing measurement is of order

(21) $$\begin{array}{c} O\left(\overset{3}{\underset{L=1}{\sum }}(\frac{I_{1}}{2^{L}}\times M_{\text{L}{\text{H}}_{L}}^{}+\frac{I_{1}}{2^{L}}\times M_{\text{H}{\text{L}}_{L}}^{}+\frac{I_{1}}{2^{L}}\times M_{\text{H}{\text{H}}_{L}}^{})\right). \end{array}$$

The summation complexity of sensing measurement sensing measurement is of order

(22) O(∑L=13(I12L×(MLHL−1)+I12L×(MHLL−1)+I12L×(MHHL−1))).

The following compression times are only evaluations since our FPGA implementation of the posttransform TD is not optimized. These evaluations are based on the lossy compression of an image of size 3072 × 128 at 1.0 bpp on a FPGA EVM board with system clock frequency at 88 MHz.  Table 3 demonstrates the comparison of complexity between the proposed multispectral compression algorithm and others.

From Table 3, the processing time of our algorithm reaches 0.016 us/sample, and the data throughput is 62.5MSPS, which is higher than JPEG2000 and CCSDS-IDC, so our approach has the low complexity. In our project, the space CCD camera works at an orbits altitude of 500 km, scroll angle of −40°~+40°, latitudes of −70°~+70°, and line working frequency of 7.2376 kHz~3.4378 kHz. In the line working frequency, capturing the image of 128 × 3072 requires 70.74 ms. Using our approach, the compressed image of 128 × 3072 requires 7.86 ms. So, our approach can process the four-band images simultaneously. This meets the requirement of the project.

In addition, we use the XC2V6000-6FF1152 FPGA to implement the proposed algorithm. The design language is Verilog HDL, the development platform is ISE8.2, and synthesis tool is XST.  Table 4 demonstrates the occupancy of resources of our approach.

From Table 4, the LUT occupies 67%, slices occupy 70%, and BRAM occupies 80%. Various indicators are lower than 95%, which meet the requirement of our project.

## 4. Conclusion

In this paper, we proposed a low-complexity posttransform coupled with compressing sensing (PT-CS) compression approach for remote sensing image. First, the DWT is applied to the remote sensing image. Then, a pair base posttransform is applied to the DWT coefficients. The pair base are DCT base and Hadamard base, which can be used on the high and low bit-rate, respectively. The best posttransform is selected by the *l*_*p*_ *-*norm-based approach. The posttransform is considered as the sparse representation stage of CS. The posttransform coefficients are resampled by sensing measurement matrix. Experimental results on on-board TDI-CCD camera images show that the proposed approach significantly outperforms the CCSDS-IDC-based coder, and its performance is comparable to that of the JPEG2000 at low bit rate and it does not have the high excessive implementation complexity of JPEG2000.

## Figures and Tables

**Table 1:** Input date rates of compression system for France earth observation satellites in recent years.

Satellite	SPOT4 (1998)	SPOT5 (2002)	PLEIADES (2010)

Coverage width	60	60	20

Resolution	10	2.5	0.7

Data rates	32 Mb/s	128 Mb/s	4.5 Gb/s

**Table 2:** Test results of compression.

Images	PSNR (dB)	Average PSNR (dB)	Bit rate (bits/pixel)
SPOT-1	41.32	40.62	0.25
SPOT-2	41.39		
SPOT-3	39.17		

SPOT-1	42.92	42.26	0.5
SPOT-2	43.06		
SPOT-3	40.81		

SPOT-1	42.75	44.21	1
SPOT-2	45.11		
SPOT-3	44.78		

SPOT-1	43.97	45.44	2
SPOT-2	46.32		
SPOT-3	46.04		

**Table 3:** The results of complexity comparison.

Methods	Times
Ours	0.016 us/sample
JPEG2000 [13]	0.11 us/sample
JPEG2000 [14]	0.04 us/sample
CCSDS-IDC [15]	0.08 us/sample
CCSDS-IDC [16]	0.05 us/sample
CCSDS-IDC [17]	0.025 us/sample

**Table 4:** The occupancy of resources.

Resources	Utilization rate
Slices	23655/33792 (70%)
4 input LUTs	45281/67584 (67%)
BRAM	116/144 (80%)
